# Tumour extracellular vesicle‐derived Complement Factor H promotes tumorigenesis and metastasis by inhibiting complement‐dependent cytotoxicity of tumour cells

**DOI:** 10.1002/jev2.12031

**Published:** 2020-11-28

**Authors:** Xiaowen Mao, Longyin Zhou, Sze Keong Tey, Angel Po Yee Ma, Cherlie Lot Sum Yeung, Tung Him Ng, Samuel Wan Ki Wong, Bonnie Hei Man Liu, Yi Man Eva Fung, Edward F. Patz, Peihua Cao, Yi Gao, Judy Wai Ping Yam

**Affiliations:** ^1^ Department of Pathology, Li Ka Shing Faculty of Medicine The University of Hong Kong Pokfulam Hong Kong; ^2^ Department of Chemistry, State Key Laboratory of Synthetic Chemistry The University of Hong Kong Pokfulam Hong Kong; ^3^ Department of Radiology Duke University Medical Center Durham USA; ^4^ Department of Pharmacology and Cancer Biology Duke University Medical Center Durham USA; ^5^ Clinical Research Center, Zhujiang Hospital Southern Medical University Guangzhou Guangdong P. R. China; ^6^ Department of Hepatobiliary Surgery II, Zhujiang Hospital Southern Medical University Guangzhou Guangdong P. R. China; ^7^ Institute of Regenerative Medicine, Zhujiang Hospital Southern Medical University Guangzhou P. R. China; ^8^ Artificial Organs and Tissue Engineering Centre of Guangdong Province Guangzhou P. R. China; ^9^ State Key Laboratory of Organ Failure Research Southern Medical University Guangzhou P. R. China; ^10^ State Key Laboratory of Liver Research (The University of Hong Kong) Pokfulam Hong Kong

**Keywords:** Complement Factor H, complement‐mediated cytotoxicity, extracellular vesicles, hepatocellular carcinoma

## Abstract

The complement system is involved in the immunosurveillance of pathogens and tumour cells. Proteomic profiling revealed that extracellular vesicles (EVs) released by metastatic hepatocellular carcinoma (HCC) cells contained a significant number of complement proteins. Complement Factor H (CFH), an abundant soluble serum protein that inhibits the alternative complement pathway, was found to be highly expressed in EVs of metastatic HCC cell lines. Here, we investigated the functional role of EV‐CFH and explored the therapeutic efficacy of targeting EV‐CFH with an anti‐CFH antibody in HCC. The results showed that EVs that are enriched in CFH promoted HCC cell growth, migration, invasiveness and enhanced liver tumour formation in mice. EV‐CFH also promoted metastasis, which was significantly abrogated when treated with an anti‐CFH antibody. These findings demonstrate an unexplored function of EV‐CFH in protecting HCC cells by evading complement attack, thereby facilitating tumorigenesis and metastasis. Lastly, we demonstrated the therapeutic efficacy of an anti‐CFH antibody in suppressing tumour formation in a syngeneic mouse model. This study suggests a new therapeutic strategy for HCC, by inhibiting EV‐CFH with a tumour specific anti‐CFH antibody.

## 1 INTRODUCTION

The immune system has the ability to recognize and distinguish cancer cells from normal cells, leading to an anti‐tumour response (Okabe & Medzhitov, 2014). The complement system links the innate and adaptive immune responses. It is a major component of the immune system that protects the host by eliminating pathogens and cells that are damaged or altered (Walport, 2001a, 2001b). It also elicits inflammatory responses and maintains host homeostasis (Merle, Noe, Halbwachs‐Mecarelli, Fremeaux‐Bacchi, & Roumenina, 2015). Three different complement pathways, namely, the classical pathway, the lectin pathway and the alternative pathway, all converge to activate the central component C3 by different mechanisms leading to the release of anaphylatoxins, C3a and C5a, and the subsequent assembly of complement membrane attack complex (MAC) to protect the host (Xie, Jane‐Wit, & Pober, 2020). The complement components are composed of plasma proteins, cell surface receptors and intracellular proteins (Reis, Mastellos, Ricklin, Mantovani, & Lambris, 2018). The complement proteins, which are expressed by cancer cells, stromal cells and immune cells in the tumour microenvironment work together to affect the fate of the cancer cells. Recent findings suggest the significance of complement proteins in tumour formation and cancer metastasis. Both pro‐tumoural and anti‐tumoural roles of individual complement proteins have been demonstrated in cell lines and mouse models of different cancer types. Leptomeningeal metastatic cells secrete complement C3 which has been proven to promote cancer growth in cerebrospinal fluid (Boire et al., 2017). In a mouse model of breast cancer, C5a receptor 1 facilitates metastasis by inducing immunosuppressive response in the lungs (Vadrevu et al., 2014). On the contrary, the production of intra‐tumoural anaphylatoxins induced by radiotherapy is crucial for tumour‐specific immunity and therapeutic efficacy (Surace et al., 2015). These findings indicate that the functions of complement proteins in cancer growth and metastasis are highly context dependent (Roumenina, Daugan, Petitprez, Sautès‐Fridman, & Fridman, 2019).

Cancer cells formulate evasion methods to create an inhibitory mechanism, thereby reducing complement‐mediated cytotoxicity. One mechanism by which cancer cells can evade complement attack is through the release of EVs. EVs mediate intercellular communications via the transfer of its components to neighbouring and distant cells. The uptake of EVs by the recipient cells influences their signalling network and phenotypes. Tumour‐derived EVs have garnered much attention for their abilities to promote an aggressive phenotype (Al‐Nedawi et al., 2008; Kogure, Lin, Yan, Braconi, & Patel, 2011; Liu, Fan, & Yu, 2019), modulate the local tumour microenvironment (Fang et al., 2018; Paggetti et al., 2015), facilitate the formation of pre‐metastatic niche in distant organ sites (Costa‐Silva et al., 2015; Peinado et al., 2012; Rodrigues et al., 2019) and inhibit complement‐dependent cytotoxicity towards cancer cells (Capello et al., 2019). There is increasing evidence that EVs shed by various cell types are loaded with key complement regulators (Karasu, Eisenhardt, Harant, & Huber‐Lang, 2018). Complement regulators including CD55, C3, and C9 have been reported in EVs derived from human colon cancer cells (Mathivanan et al., 2010), melanoma cells (Peinado et al., 2012) and ovarian cancer cells (Liang et al., 2013). However, the functional implications of these proteins in cancers are yet to be explored. A study reported that in response to complement attack, mortalin/GRP75 is released in association with MAC‐loaded EVs from erythroleukaemic cells and defend the cells from complement‐mediated cytotoxicity (Pilzer & Fishelson, 2005). Another study revealed that the release of vesicles with complement C9 by oligodendrocytes is observed during the recovery from complement‐mediated cell injury (Scolding et al., 1989). Collectively, these studies provide insights into how EVs secreted by cells participate in the regulation of the immune response.

In this study, we found that Complement Factor H (CFH), a key inhibitor of the alternative pathway, was enriched in EVs of metastatic HCC cells. In mice educated with EVs of metastatic non‐target control HCC cells, liver tumour development was significantly accelerated when compared to mice without EV injection. However, such enhancement was abrogated in mice when injected with EVs with reduced CFH derived from CFH knockdown cells. Our findings also demonstrated the role of EV‐CFH in promoting metastasis in mice. EV‐CFH was shown to play a role in protecting cells from complement‐mediated cell lysis. In a syngeneic mouse model, anti‐CFH antibody significantly suppressed tumour development. These findings suggest that the modulation of the complement system in patients with EV‐CFH can be potentially exploited as a therapeutic strategy in HCC.

## 2 MATERIAL AND METHODS

### 2.1 Cell culture

Human HCC cell lines Hep3B, Huh7 and PLC/PRF/5, human 293FT and murine Hepa1‐6 cell lines were purchased from the American Type Culture Collection (ATCC). HLE was obtained from the Japanese Collection of Research Bioresources (JRCB), and two metastatic HCC cell lines MHCC97L and MHCCLM3 were obtained from the Cancer Institute, Fudan University, China (Sun et al., 1996). MHCC97L was stably infected with lentiviral‐based luciferase plasmid for in vivo bioluminescence imaging. H2P and H2M were kindly provided by Professor XY Guan, The University of Hong Kong (Hu et al., 2004). The immortalized normal liver cell line MIHA was provided by Jayanta Roy‐Chowdhury, Albert Einstein College of Medicine, New York (Brown et al., 2000). Murine p53 null hepatoblasts transduced by Myc (p53‐/‐;Myc hepatoblasts) were provided by Scott Lowe, Memorial Sloan Kettering Cancer Center, New York (Xue et al., 2012). All cells lines were cultured according to the provider's recommendations and were routinely tested to avoid mycoplasma contamination. Authentication of HCC cell line identity was performed by short tandem repeat profiling (Pangenia Lifesciences Ltd).

### 2.2 Establishment of CFH knockdown clones

The non‐metastatic HCC Huh7 and metastatic HCC MHCC97L cell lines were used to establish non‐target control (CTL‐KD) and CFH knockdown stable clones (CFH‐KD1 and CFH‐KD2) using MISSION non‐target shRNA control vector and two different shRNA targeting sequences of CFH (Sigma‐Aldrich), sh‐57134 (5′‐CCGGGCCAGTAATGTAACATGCATTCTCGAGAATGCATGTTACATTACTGGCTTTTTG‐3′) and sh‐371774 (5′‐CCGGTACTCACCTTTAAGGATTAAACTCGAGTTTAATCCTTAAAGGTGAGTATTTTTG‐3′), respectively. 293FT cells were co‐transfected with shRNA, Lenti‐Pac HIV mix (GeneCopoeia Inc.) and EndoFectin Lenti transfection reagent (GeneCopoeia Inc.). The viral supernatant was collected and used to transduce HCC cells. The transduced cells were selected by puromycin (Merck). The CFH expression of the resistant clones was examined by western blotting.

### 2.3 Isolation and validation of EVs

For EV purification from the conditioned medium of cell cultures, 5 × 10^7^ cells were seeded in 30 ml media supplemented with 10% EV‐depleted fetal bovine serum (FBS) in a 15‐cm tissue culture plate for 72 h. EVs were depleted from FBS by overnight centrifugation at 100,000 × *g* at 4°C (Beckman Coulter, Avanti JXN‐30). EVs were isolated from the conditioned medium by a standard differential centrifugation protocol. Briefly, the conditioned medium was subjected to sequential centrifugation steps of 3000 × *g* for 15 min and 20,000 × *g* for 30 min to remove cells and cellular debris. The resulting conditioned medium was filtered using a 0.22 μm filter (Millipore) to eliminate large cell debris and membranes. A pellet was collected after 2 h of ultracentrifugation at 100,000 × *g* using JA‐30.50Ti fixed angle rotor (Beckman Coulter) and was resuspended in PBS for washing. The resulting EV pellet was collected after 70 min ultracentrifugation at 100,000 × *g* and resuspended in PBS for downstream analyses. Due to the limited volume of serum obtained from mice, the purification of circulating EVs from mouse serum was performed using the ExoQuick PLUS Exosome Purification Kit for Serum & Plasma (System Biosciences). Mouse serum of 250 μl in volume was first centrifuged at 16,500 × *g* for 45 min (Eppendorf, 5430R) to pellet large vesicles. EVs were then purified using the purification kit according to the manufacturer's protocol.

The amount of EVs were quantified using Bradford reagent (Bio‐Rad Corporation), with bovine serum albumin at a standard concentration. The particle number of EVs was determined using ZetaView BASIC NTA PMX‐120 (Particle Metrix GmbH). To validate the isolated EVs, the morphology of EVs was visualized using Philips CM100 Transmission Electron Microscope (FEI Company). The size range of EVs was analysed by ZetaView BASIC NTA PMX‐120 (Particle Metrix GmbH). The identity of EVs was revealed by western blotting using antibodies against positive (TSG101, Alix, CD9) and negative (nucleoporin p62, cis‐Golgi marker GM130) EV markers. We have submitted all relevant data of our experiments to the EV‐TRACK knowledgebase (EV‐TRACK ID: EV200083) (Consortium, Van Deun, & Mestdagh, 2017).

### 2.4 Proteomic analysis by mass spectrometry

Protein lysates of EV in 8 M urea/100 mM Tris‐HCl buffer was incubated at 60°C for 10 min. Dithiothreitol (DTT) was then added to the samples at a final concentration of 5 mM and incubated for 20 min at room temperature. Then iodoacetamide was added to a final concentration of 25 mM and incubated in the dark for 30 min. Subsequently, trypsin was added at a ratio of 1:50 (trypsin:protein) after dilution of buffer to 1 M of urea and incubated at 37°C for 16 h. The proteolysis was quenched by the addition of 5% formic acid. The digested samples were desalted using C18 STAGE tips and concentrated by SpeedVac (Thermo Savant).

The digested protein samples were analysed with HPLC‐MS/MS. The analytical column was a self‐packed PicoTip column (360 μm outer diameter, 75 μm inner diameter, 15 μm tip, New Objective) packed with 10 cm length of C18 material (ODS‐A C18 5‐μm beads, YMC) using a high‐pressure injection pump (Next Advance). The mobile phases consisted of A (0.1% formic acid) and B (0.1% formic acid in ACN). Each sample (containing 2 μg peptides) was loaded onto the analytical column by the auto‐sampler and rinsed with 2% B for 6 min at a flow rate of 500 nl/min, and subsequently eluted with a linear gradient of B from 2% to 40% for 120 min at a flow rate of 200 nl/min.

For the mass spectrometry analysis, LTQ‐Orbitrap Velos mass spectrometer (Thermo Fisher Scientific) was operated in a data‐dependent mode cycling through a high‐resolution (6000 at 400 *m*/*z*) full scan MS1 (300 ‐ 2000 *m*/*z*) in Orbitrap followed by CID MS2 scans in LTQ on the 20 most abundant ions from the immediately preceding full scan. The selected ions were isolated with a 2‐Da mass window and put into an exclusion list for 60 s after they were first selected for CID.

Raw files generated during LC‐MS/MS analysis were searched against the Uniprot Human database with MaxQuant search engine (version 1.5.5.1), in which the search was specified to trypsin digestion (allowed up to two missed cleavages), oxidation of methionine as a dynamic modification, and iodoacetamide derivative of cysteine as a static modification. The mass tolerance for MS1 was 20 ppm for first search and 4.5 ppm for main search, and that for MS2 was 0.5 Da. With a decoy search strategy, the peptide false discovery rate (FDR) was set to 1%. Label‐free quantification (LFQ) option was enabled with normalization. Proteins with non‐zero LFQ intensities in ≥2 out of three replicates were considered as presence in EVs while proteins with zero LFQ intensities in ≥2 out of three replicates were considered as absence in EVs. Proteins that were expressed in EVs of MHCC97L cells were subjected to analysis using DAVID v6.8 pathway program (KEGG_PATHWAY).

### 2.5 Treatment of cells with EVs for functional assays

Cells pretreated with EVs collected from the conditioned medium of control and CFH knockdown cells were functionally characterized. For all in vitro assays, 2 × 10^6^ cells were treated with 30 μg of EVs for 72 h prior to functional characterization. The detailed description of in vitro assays was included in the Supplementary Materials and Methods.

### 2.6 EV education model

Male 6‐week‐old BALB/cAnN‐nu mice were injected intravenously with 15 μg of EVs or PBS once per week for 3 weeks prior to orthotopic liver implantation. The tumour seed for orthotopic liver implantation was obtained by subcutaneous injection of luciferase‐labelled MHCC97L cells in BALB/cAnN‐nu mice. Tumours were resected and cut into 1 mm^3^ cubes which were implanted in the liver lobes of mice. Five mice were used in each experimental group. Six weeks after implantation, the in vivo tumour formation in the liver was determined by bioluminescence imaging using IVIS 100 Imaging System (Xenogen) after intraperitoneal injection of D‐luciferin (Xenogen). All the animal experimentations were performed according to the Animals (Control of Experiments) Ordinance (Hong Kong) and the Institute's guidance from Laboratory Animal Unit on animal experimentation was closely followed.

### 2.7 Experimental metastasis mouse model

For each mouse, 1×10^5^ murine p53‐/‐;Myc hepatoblasts with 10 μg EVs derived from the MHCC97L control or CFH knockdown cells dissolved in 100 μl PBS were injected intravenously into male 5–6 weeks old BALB/c nude mice. Five mice were used in each group. Bioluminescence imaging was performed 14 days post injection. The number of cells colonized in the lungs was estimated by the intensity of the bioluminescence signals. At the end of the experiment, mice were sacrificed and lung tissues were excised for histological analysis.

### 2.8 Anti‐CFH antibody treatment

The neutralizing effect of anti‐human CFH antibody on the biological activity of EVs derived from metastatic MHCC97L and their eventual effect on MIHA and PLC/PRF/5 was assessed. The antibody, which specifically targeted membrane‐bound CFH, was used in the study (Bushey et al., 2016). Cells were treated with 100 μg EVs in the presence and absence of 250 μg/ml anti‐CFH antibody. Cells treated with the same amount of control IgG were included as a control. After 72 h of incubation, cells were subjected to migration and invasion assays. The readout was compared between cells treated with or without EVs, and between EVs‐treated cells with or without anti‐CFH antibody. To assess the effect of anti‐CFH antibody on tumorigenesis, BALB/cAnN‐nu mice were injected intravenously with PBS or 15 μg MHCC97L EVs with/without IgG or anti‐CFH antibody for 3 weeks before subjected to orthotopic liver implantation of luciferase‐labelled MHCC97L tumour seed. Bioluminescence imaging was performed 6 weeks after implantation. To assess the effect of anti‐CFH antibody on EV‐induced colonization of cells in the lungs, 1 × 10^5^ murine p53‐/‐;Myc hepatoblasts and 10 μg EVs of MHCC97L, together with IgG or anti‐CFH antibody, were injected into mice though the tail vein. Four mice were used in each group. Bioluminescence imaging was performed 14 days post injection. The excised lung tissues were subjected to histological analysis.

### 2.9 Syngeneic mouse liver cancer model

The therapeutic efficacy of monoclonal anti‐mouse CFH antibody (mAb7968) (Bushey et al., 2016) in the growth of tumours derived from murine Hepa1‐6 hepatoma cells was tested in C57BL/6N mice. Cells of 1 × 10^6^/0.1 ml per site were injected subcutaneously into the mice. When the tumours reached around 5 mm in diameter, administration of anti‐CFH antibody (200 μg/0.1 ml/mouse) by intraperitoneal injection was started once every 3 days for a total of 2 weeks. Mice injected with PBS or mouse IgG antibody were performed as control experiments. Five mice were used in each group. Development of tumour growth was monitored regularly by measuring the length and width of the tumours with a calliper. The tumour volume was calculated according to the formula, 0.5 × length × width^2^. All mice were sacrificed at the end of the experiment, with their livers and blood sera harvested for further analysis.

### 2.10 Analysis of anaphylatoxin level in the conditioned medium

The levels of anaphylatoxins C3a and C5a were measured as an indicator of complement activation. Cells were incubated with 1:8 diluted normal human serum (NHS) (Complement Technology) which was used as a source of complement. After 4 h of incubation, the conditioned medium was collected and measured for C3a and C5a levels using Complement C3a Human ELISA Kit (Thermo Fisher) and Human Complement C5a ELISA Kit (Abcam), respectively. All measurements were performed in triplicates.

### 2.11 Cytotoxicity assay

Cell cytotoxicity assay was performed using CytoTox96 Non‐Radioactive Cytotoxicity Assay (Promega) which quantitatively measured lactate dehydrogenase (LDH), a stable cytosolic enzyme released upon cell lysis. Normal human serum (NHS), which is a source of complement, was added at 1:40 dilution to cells already pretreated with EVs for 72 h. After incubation for 16 h, 5 × 10^3^ PLC cells or 1 × 10^4^ MIHA cells per well were seeded in triplicates in a 96‐well plate. After incubation at 37°C for 10 h, the level of LDH released into the culture medium was measured with a 30‐min coupled enzymatic assay. The absorbance reading at 490 nm was measured by Infinite F200 microplate reader (Tecan).

### 2.12 Immunofluorescent staining

To examine the formation of the membrane attack complex in cells treated with EVs under NHS‐induced complement activation, PLC/PRF/5 cells were seeded on coverslips and incubated with EVs for 72 h followed by NHS stimulation. The EV‐treated cells were fixed with 4% paraformaldehyde in PBS, permeabilized with 0.2% Triton‐X‐100 in PBS and blocked with 3% bovine serum albumin in PBS at room temperature. The cells were then subjected to incubation with 1:500 anti‐C5b‐9 antibody (Abcam) and subsequent incubation with 1:200 Goat anti‐Rabbit IgG (H+L) Cross‐Adsorbed Secondary Antibody, Alexa Fluor 488 (Thermo Fisher) and DAPI (4′,6‐diamidino‐2‐phenylindole, dihydrochloride) (Thermo Fisher). The processed coverslips were mounted in Vectashield anti‐fade mounting medium (Vector Laboratories). Images were captured by ZEISS LSM 900 confocal microscope (Carl Zeiss).

### 2.13 Statistical analysis

All in vitro assays were performed in triplicates and three independent experiments were done. The data of all assays were calculated as mean ± standard error of mean (SEM). Student's *t*‐test and one‐way ANOVA performed by GraphPad Prism 6 were used for the statistical analysis. The major molecular function of MHCC97L EV proteins was analysed using DAVID v6.8 pathway program (KEGG_PATHWAY). *P* < 0.05 was considered statistically significant.

## 3 RESULTS

### 3.1 EVs derived from metastatic HCC cells display potent promoting effect in cell motility and invasion

**FIGURE 1:**
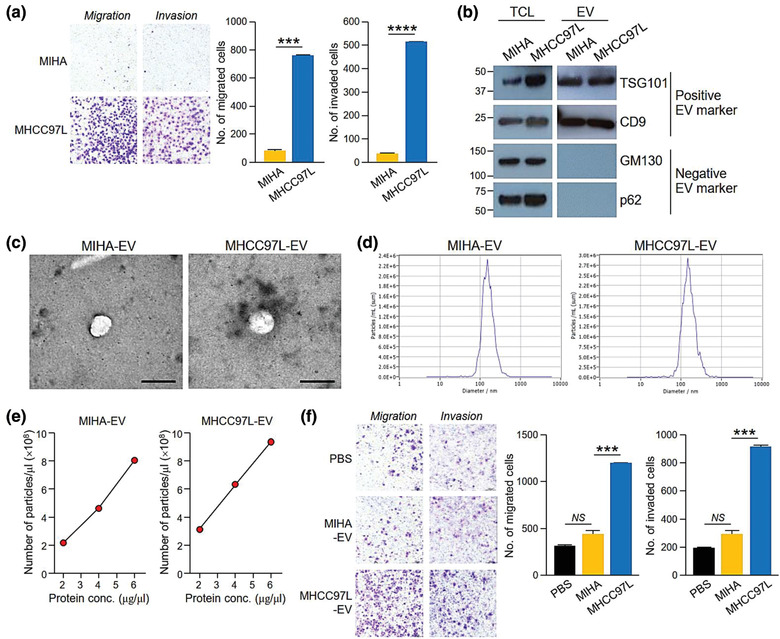
EVs of metastatic HCC cells displayed promoting effect in cell migration and invasion. (a) MIHA and MHCC97L were seeded at 4 × 10^4^ cells per well in triplicates for migration and invasion assays. After incubation, the migrated and invaded cells were fixed, stained and counted. Representative images of cells are shown. The number of cells is plotted. (b) Western blotting of EV molecular markers in 30 μg of total cell lysate (TCL) of MIHA and MHCC97L cells, and 15 μg of EVs derived from these 2 cell lines. Positive EV markers include TSG101 and CD9 while negative EV markers are *cis*‐Golgi marker GM130 and nucleoporin p62. (c) Representative electron micrographs of EVs collected from the conditioned medium of MIHA and MHCC97L cells evaluated are shown. Scale bar, 200 nm. (d) Size distribution of MIHA‐ and MHCC97L‐EVs were measured by nanoparticle tracking analyser. (e) Amount of EVs was determined in terms of number of particles and protein amount using nanoparticle tracking analyser and Bradford assay, respectively. Number of EVs per μl against protein concentration of EVs (μg/μl) is plotted. (f) Examination of the cell migration and invasiveness of PLC/PRF/5 cells pretreated with EVs collected from the conditioned medium of MIHA or MHCC97L cells. PLC/PRF/5 cells were seeded at 7 × 10^4^ cells per well in triplicates for the assays. Migrated and invaded cells were fixed, stained and counted. Representative images of cells are shown. The number of cells is plotted. Data are represented as mean ± SEM. ^*^ *P* < 0.05, ^**^ *P* < 0.01, ^***^ *P* < 0.001, *NS*, not significant. *P* < 0.05 is considered as statistically significant

Migratory and invasive potentials of cancer cells are critical determinants for the success of metastasis. Compared to the immortalized normal liver cell line MIHA, metastatic MHCC97L cells displayed a significantly higher capacity to migrate and invade (Figure 1a). Apart from the intrinsic properties of cancer cells, distant communication between tumour cells and their microenvironment has been recognized to play a crucial role during cancer development and metastasis. EVs, key mediators of cell‐to‐cell communication, were collected from the conditioned medium of MIHA and MHCC97L cells. From 72‐h cultures of 5 × 10^7^ MIHA and MHCC97L cells, 10 and 50 μg of EVs were obtained, respectively. The collected EVs were shown to express positive markers but not negative markers (Figure 1b). The electron micrograph showed the spherical morphology of EVs (Figure 1c) and the peak size of EVs was around 130–140 nm (Figure 1d). According to MISEV2018, the EVs derived from both MIHA and MHCC97L cells fell within the size range of medium and/or large EVs. It was noted that 1 μg of intact EVs was equivalent to 1.475–1.575 × 10^8^ number of EVs (Figure 1e). The effect of EVs from MIHA and MHCC97L cells on PLC/PRF/5 cells showed that MHCC97L‐EVs but not MIHA‐EVs significantly promoted migration and invasion of PLC/PRF/5 cells (Figure 1f).

### 3.2 CFH is highly expressed in EVs of metastatic HCC cells

**FIGURE 2:**
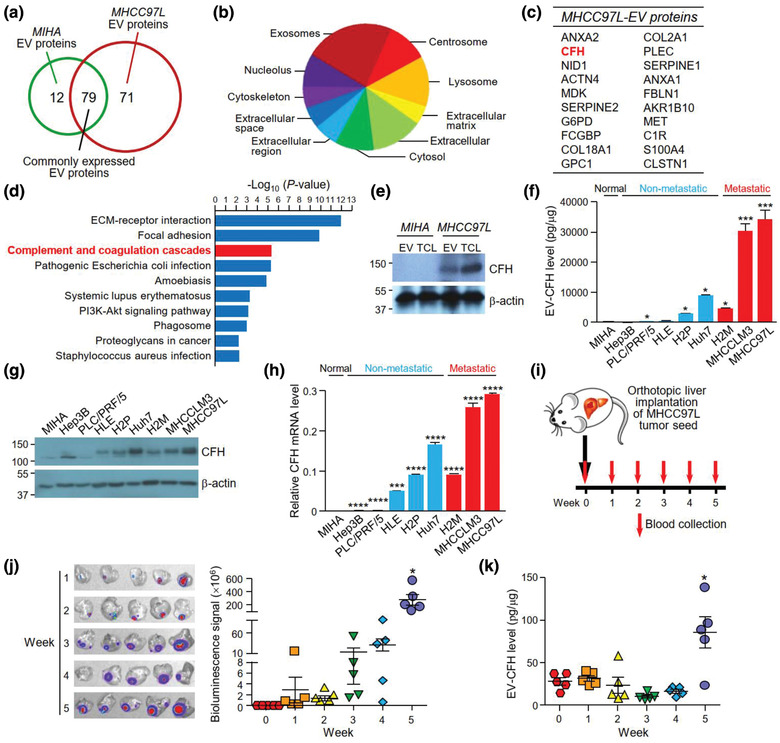
CFH is identified in EVs of metastatic HCC cells. (a) The Venn diagram illustrates the number of proteins expressed in either or both EVs of MIHA or MHCC97L cells. (b) Analysis of the distribution of cellular components of proteins detected in MHCC97L‐EVs using FunRich3.1.3. (c) Top 20 unique proteins identified in MHCC97L‐EVs are listed according to their expression intensity as detected by mass spectrometry. (d) Pathway analysis of proteins found in MHCC97L‐EVs using DAVID v6.8 pathway program. (e) Analysis of CFH expression in 30 μg of total cell lysate (TCL) and 15 μg of EVs of MIHA and MHCC97L cells by western blot analysis. (f) Analysis of CFH expression in EVs derived from various HCC cell lines by ELISA. Non‐metastatic and metastatic cell lines are indicated by blue and red colours, respectively. Protein (g) and mRNA (h) expressions of CFH were analysed by immunoblotting and quantitative PCR, respectively. The levels of EV‐CFH and CFH mRNA of HCC cell lines were statistically compared with that of MIHA cell line. (i) Blood was obtained from mice before and weekly after orthotopic liver implantation of luciferase‐labelled MHCC97L tumour seed (n = 5). EVs were isolated from the serum. (j) *Ex vivo* bioluminescence imaging of liver tissues. Quantification of the luminescence intensity is shown. (k) Analysis of CFH level in the circulating EVs (EV‐CFH) isolated from the serum of mice using ELISA. The bioluminescence signal and EV‐CFH level were statistically analysed with reference to week 0. Data are represented as the mean ± SEM; ^*^ *P* < 0.05. *P* < 0.05 is considered as statistically significant

To interrogate the differential biological activity of MIHA‐ and MHCC97L‐EVs, the proteomic profiles of their EVs were determined and compared using mass spectrometry (Table S1). Proteins with FDR less than 1% were analysed. A total of 79 proteins were found in EVs of both MIHA and MHCC97L cells, while 12 and 71 proteins were only detected in EVs of MIHA and MHCC97L cells, respectively (Figure 2a, Table S2). FunRich software was employed to analyse the cellular distribution of EVs proteins identified in MHCC97L cells. Proteins of MHCC97L‐EVs were mainly exosomal proteins (Figure 2b). The top 20 proteins uniquely expressed in MHCC97L‐EVs were listed based on their abundance and shown in Figure 2c. Pathway analysis found that proteins of MHCC97L‐EVs were significantly associated with major molecular pathways such as extracellular matrix‐receptor interaction, focal adhesions and complement and coagulation cascades (Figure 2d, Table S3). Concomitantly, several complement related proteins were either uniquely expressed or upregulated in MHCC97L‐EVs when compared to MIHA‐EVs (Tables S4 and S5). Based on the potential role of EV proteins on the complement system, CFH, which was the most abundant complement protein on the list, was selected for further analysis. The differential expression of CFH in the total cell lysates and EVs of MIHA and MHCC97L cells was validated (Figure 2e). Accordingly, immunogold labelling also revealed higher CFH expression on the surface of MHCC97L‐EVs than that of MIHA‐EVs (Figure S1). We further extended the analysis of EV‐CFH in a range of HCC cell lines (Figure 2f). Compared with EVs of non‐metastatic cells, CFH level was higher in EVs of metastatic cells. In agreement with EV‐CFH level, the protein and transcript levels of CFH were generally higher in metastatic cells than non‐metastatic cells (Figure 2g‐h). To investigate whether CFH could be detected in the circulating EVs of animals, tumour seeds derived from luciferase‐labelled MHCC97L cells were orthotopically implanted into the liver of mice (Figure 2i). Bioluminescence signal revealed the progressive development of liver tumour (Figure 2j). Circulating EVs were collected weekly for the analysis of EV‐CFH (Figure 2k, Figure S2A‐S2C). It was noted that the EV‐CFH level displayed a significant elevation at week 5 post implantation. These data suggest that the level of EV‐CFH correlates with the tumour development in mice.

### 3.3 EVs with reduced CFH level revealed decreased promoting activity in HCC cell growth and motility

**FIGURE 3:**
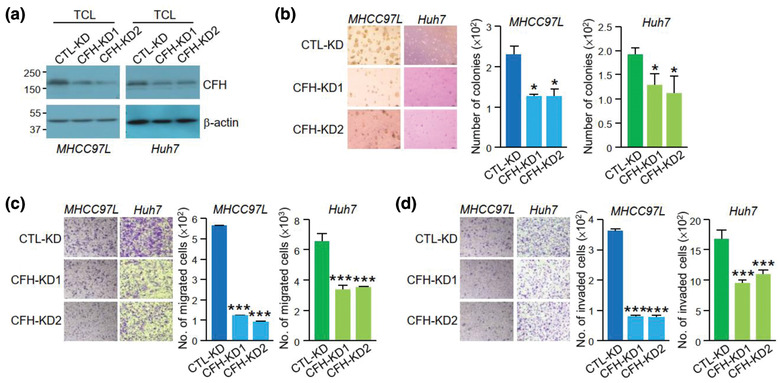
CFH promotes HCC cell anchorage independent growth, migration and invasion. (a) Immunoblotting showing CFH expression in the total cell lysate (TCL) of non‐target control (CTL‐KD) and CFH knockdown clones (CFH‐KD1 and CFH‐KD2) established in MHCC97L and Huh7 cells. Stable non‐target control and CFH knockdown cells were subjected to soft agar (b), migration (c) and invasion (d) assays. Readout of CFH knockdown cells was compared with the readings of the corresponding control cells. Data are represented as mean ± SEM. ^*^ *P* < 0.05, ^***^ *P* < 0.001. *P* < 0.05 is considered as statistically significant

**FIGURE 4:**
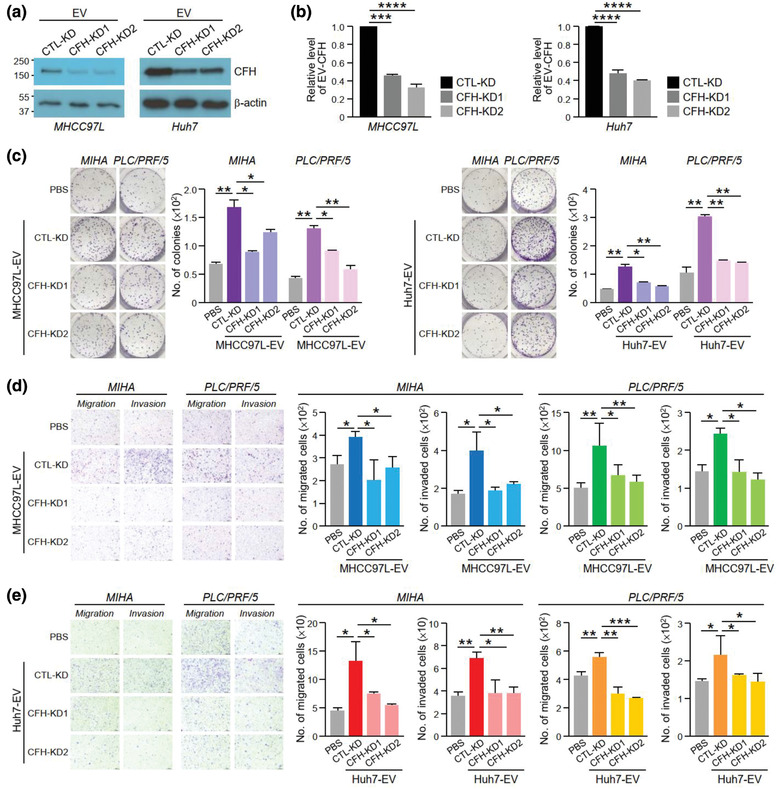
EVs with reduced CFH displayed diminished activity in promoting HCC cell growth, migration and invasiveness. Analysis of CFH level in EVs (EV‐CFH) of the control (CTL‐KD) and CFH knockdown (CFH‐KD1 and CFH‐KD2) cells established in MHCC97L (*left*) and Huh7 (*right*) cells using western blot analysis (a) and ELISA (b). Colony formation assay of MIHA and PLC/PRF/5 cells pretreated with EVs derived from MHCC97L (c) and Huh7 (d) control and CFH knockdown cells. Quantification of the number of colonies is shown. Representative image showing the fixed and crystal violet stained colonies. Examination of the cell migration and invasiveness of MIHA and PLC/PRF/5 cells pretreated with EVs of MHCC97L (e) and Huh7 (f) control and CFH knockdown cells. Representative images of fixed and crystal violet stained migrated and invaded cells at the end of the experiment are shown. Quantification of the number of cells is shown. Data are represented as mean ± SEM. ^*^ *P* < 0.05, ^**^ *P* < 0.01, ^***^ *P* < 0.001. *P* < 0.05 is considered as statistically significant

To examine the function of CFH in tumour cells, stable CFH knockdown (CFH‐KD1 and CFH‐KD2) and non‐target control cells (CTL‐KD) were established in non‐metastatic Huh7 and metastatic MHCC97L cells, both of which had relatively high levels of CFH (Figure 3a). Knockdown of CFH reduced the ability of MHCC97L and Huh7 cells to grow, migrate and invade (Figure 3b‐d). We further examined the functions of EVs derived from these stable clones. The identity, morphology and size of the isolated EVs were validated (Figure S3A‐S3C). The expression of CFH was reduced in EVs derived from CFH‐KD cells (CFH‐KD‐EVs) when compared with EVs of CTL‐KD cells (CTL‐KD‐EVs) as revealed by western blot analysis (Figure 4a) and ELISA (Figure 4b). The effect of these EVs was subsequently tested on MIHA and PLC/PRF/5 cells. Cells treated with CTL‐KD‐EVs showed enhanced aggressiveness when compared with cells treated with PBS in colony formation assay, migration and invasion assays (Figure 4c‐e). However, such enhancement was abrogated when cells were treated with CFH‐KD‐EVs, indicating that CFH plays a role in promoting the activity of EVs.

### 3.4 EV‐CFH augmented liver tumour formation and extrahepatic metastasis

**FIGURE 5:**
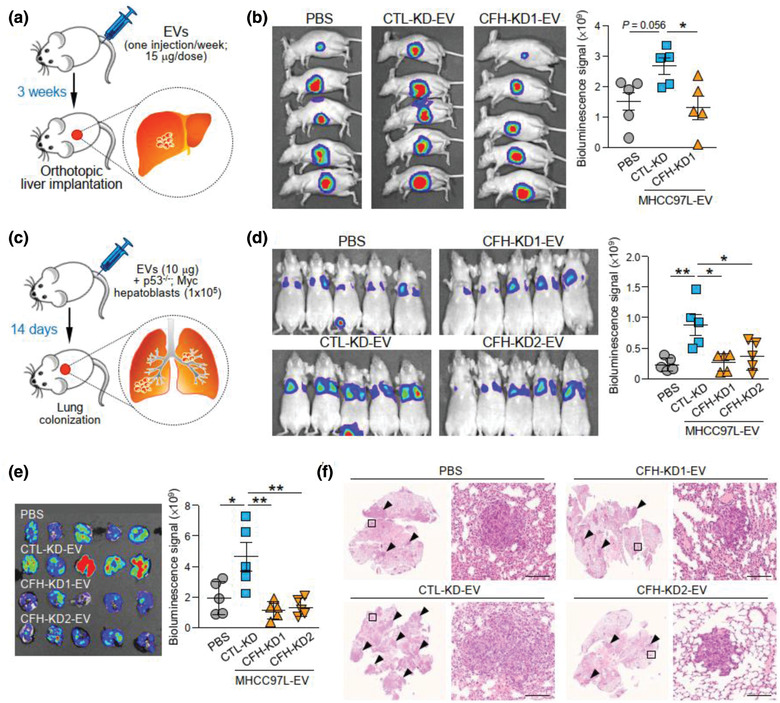
EV‐CFH promotes HCC tumorigenesis and metastasis. (a) Schematic diagram of EV education model. EVs derived from MHCC97L control (CTL‐KD) and CFH knockdown (CFH‐KD1) cells were injected into mice through tail vein once a week for 3 weeks (15 μg per week) prior to orthotopic liver implantation of luciferase‐labelled MHCC97L tumour seed (n = 5). Development of liver tumour was analysed 6 weeks after liver implantation. (b) Bioluminescence imaging of animals at the end of experiment. Quantification of luciferase signal is plotted. (c) Diagram illustrating the procedure of experimental metastasis assay. Murine p53‐/‐;Myc hepatoblasts were injected intravenously with or without EVs of MHCC97L control (CTL‐KD) or CFH knockdown (CFH‐KD1) cells (n = 5). Animals were subjected to bioluminescence imaging 14 days after injection. (d) Bioluminescence imaging of animals at the end of experiment. Intensity of luciferase signal is quantified. (e) Ex vivo bioluminescence imaging of dissected lung tissues. Quantification of luciferase signal is shown. (f) Representative images of H&E staining of lung tissues. Examples of metastatic lesions are indicated by arrowheads. Insets indicate the enlarged area of the metastatic lesions. Data are represented as mean ± SEM. ^*^ *P* < 0.05, ^**^ *P* < 0.01. *P* < 0.05 is considered as statistically significant

The role of EV‐CFH in HCC tumorigenesis was further analysed in an EV education model. Mice intravenously injected with EVs once a week for 3 weeks were orthotopically implanted with tumour seed derived from luciferase‐labelled MHCC97L cells (Figure 5a). The findings showed that the luciferase signal was enhanced in mice injected with CTL‐KD‐EVs when compared to mice injected with PBS; however, the increase in luciferase signal was not seen in mice injected with CFH‐KD‐EVs (Figure 5b). The ability of cells to colonize in distant sites determines the metastatic potential of tumour cells. The role of EV‐CFH in metastasis was studied using an experimental metastasis assay. EVs of CTL‐KD or CFH‐KD cells were injected with luciferase‐labelled murine p53‐/‐;Myc hepatocytes into mice through the tail vein (Figure 5c). Consistently, mice injected with CTL‐KD‐EVs displayed the most prominent luciferase signal when compared to mice injected with PBS or CFH‐KD‐EVs (Figure 5d). The dissected lungs exhibited a similar trend in luciferase intensity (Figure 5e). Histological analysis of lung tissues revealed a correlation between luciferase signal with the incidence of metastatic lesions (Figure 5f). These finding demonstrated the ability of EVs to promote tumour growth and metastasis by facilitating the colonization of cells in the lungs.

### 3.5 EV‐CFH inhibited complement‐dependent cytotoxicity in HCC cells

**FIGURE 6:**
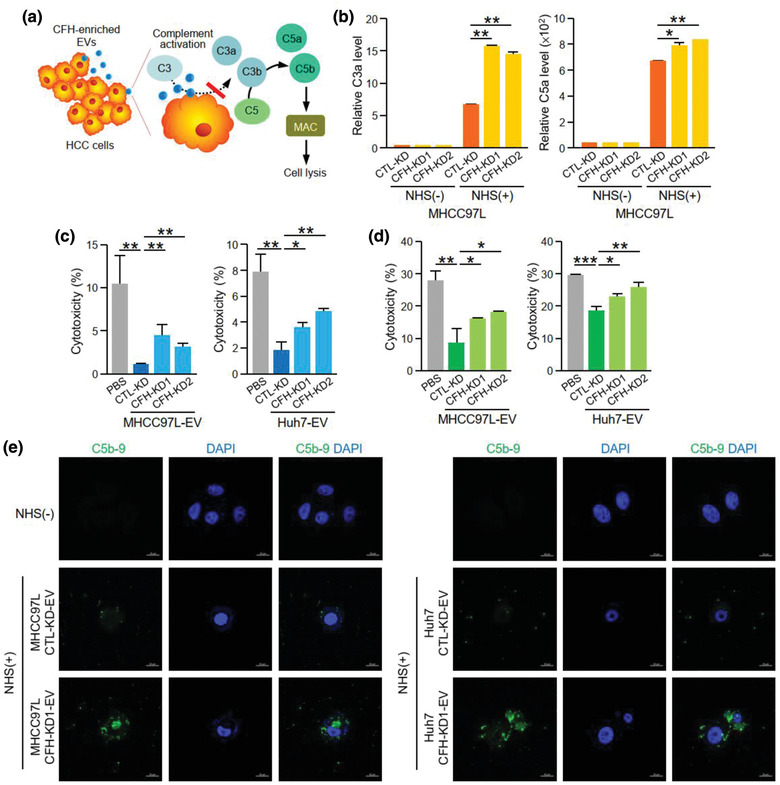
EV‐CFH inhibits complement‐dependent cytotoxicity in HCC cells. (a) Diagram showing the inhibitory effect of CFH in complement activation and formation of membrane attack complex (MAC). (b) Analysis of C3a (*left*) and C5a (*right*) levels in the conditioned medium collected from normal human serum (NHS)‐activated MHCC97L non‐target control (CTL‐KD) and CFH knockdown (CFH‐KD1 and CFH‐KD2) cells using ELISA. Analysis of cytotoxicity of MIHA (c) and PLC/PRF/5 (d) cells after treating with EVs of MHCC97L and Huh7 CTL‐KD, CFH‐KD1 and CFH‐KD2 cells under NHS stimulation. (e) The deposition of MAC on PLC/PRF/5 cells treated with CTL‐KD‐ and CFH‐KD‐EVs under NHS treatment was examined by immunofluorescent staining using anti‐C5b‐9 antibody followed by FITC‐conjugated secondary antibody. Cells were also stained with DAPI. Data are represented as mean ± SEM. ^*^ *P* < 0.05, ^**^ *P* < 0.01. *P* < 0.05 is considered as statistically significant

Activation of complement system results in the formation of a cell‐killing membrane attack complex, which protects the host. Since CFH is an inhibitor of the alternative pathway of complement activation, we asked whether MHCC97L‐EVs, which express a high level of CFH, can protect HCC cells from complement‐induced cytotoxicity. CFH inhibits complement activation by blocking the cleavage of C3 convertase to generate functional anaphylatoxins C3a and C5a (Figure 6a). To explore the potential role of CFH in tumour cells, the conditioned media of MHCC97L CTL‐KD or CFH‐KD cells activated by NHS were collected to determine the levels of C3a and C5a. As shown in Figure 5b, activation of control cells by NHS enhanced the release of C3a and C5a. When CFH was suppressed in the cells, the levels of C3a and C5a were further elevated (Figure 6b). These findings revealed the role of CFH in suppressing complement activation in HCC cells. Furthermore, MIHA and PLC/PRF/5 cells treated with CTL‐KD‐EVs showed a reduction in cytotoxicity under NHS treatment as evidenced by the reduced LDH in the conditioned medium when compared to untreated cells (Figure 6c‐d). The extent of cytotoxicity was increased when cells were treated with EVs with decreased levels of CFH. We analysed the expression of C5b‐9, the major component of membrane attack complex, in PLC/PRF/5 and Huh7 cells treated with EVs of CTL‐KD or CFH‐KD cells under complement activation. Immunofluorescent staining using anti‐C5b‐9 antibody revealed that C5b‐9 signal was only detected in cells under NHS‐stimulated cells. The intensity of C5b‐9 was reduced in cells treated with CTL‐KD‐EVs when compared to cells treated with CFH‐KD‐EVs (Figure 6e). Taken together, these results suggested that EV‐CFH protects HCC cells from complement‐mediated cytotoxicity.

### 3.6 Treatment with anti‐CFH antibody diminished the promoting effect of EVs of metastatic HCC cells

**FIGURE 7:**
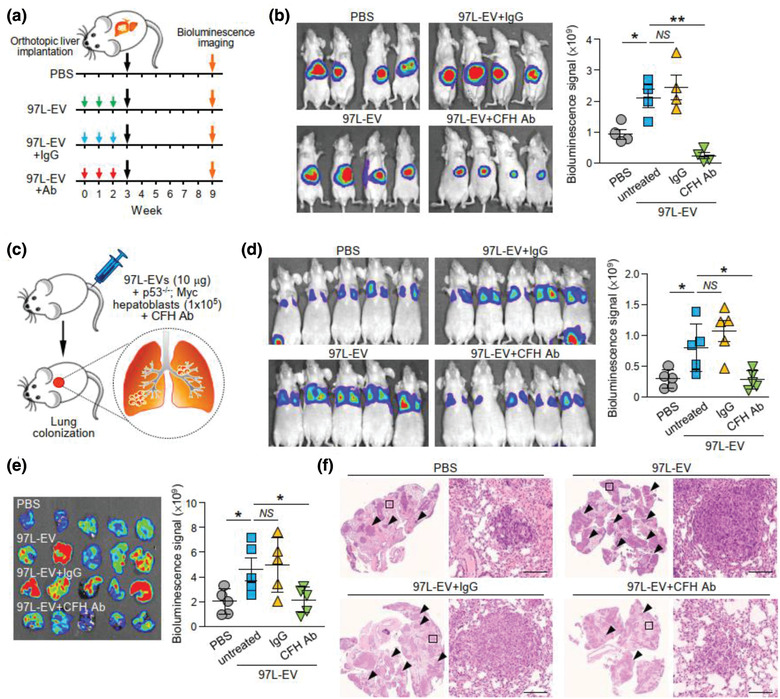
Liver tumour formation and metastasis induced by EVs of metastatic HCC cells are suppressed by anti‐CFH antibody treatment. (a) Schematic diagram of experimental procedure. EVs derived from MHCC97L cells (97L‐EV; 15 μg) were injected intravenously either alone or with IgG or anti‐CFH antibody (Ab) in mice once per week for 3 consecutive weeks followed by orthotopic liver implantation of HCC tumour seed derived from luciferase‐labelled HCC cells. Bioluminescence imaging was carried out 6 weeks after implantation. (b) Bioluminescence imaging of animals at the end of experiment. Quantification of luciferase signal is plotted. (c) Diagram illustrating the procedure of experimental metastasis assay. Murine p53‐/‐;Myc hepatoblasts were injected intravenously with or without 10 μg 97L‐EVs in the presence of IgG or anti‐CFH antibody. Animals were subjected to bioluminescence imaging 14 days after injection. (d) Bioluminescence imaging of animals at the end of experiment. Intensity of luciferase signal is quantified. (e) Ex vivo bioluminescence imaging of dissected lung tissues. Quantification of luciferase signal is shown. (f) Representative images of H&E staining of lung tissues. Examples of metastatic lesions are indicated by arrowheads. Insets show the enlarged area of the metastatic lesions. Data are represented as mean ± SEM. ^*^ *P* < 0.05, ^**^ *P* < 0.01. *P* < 0.05 is considered as statistically significant. *NS*, not significant

**FIGURE 8:**
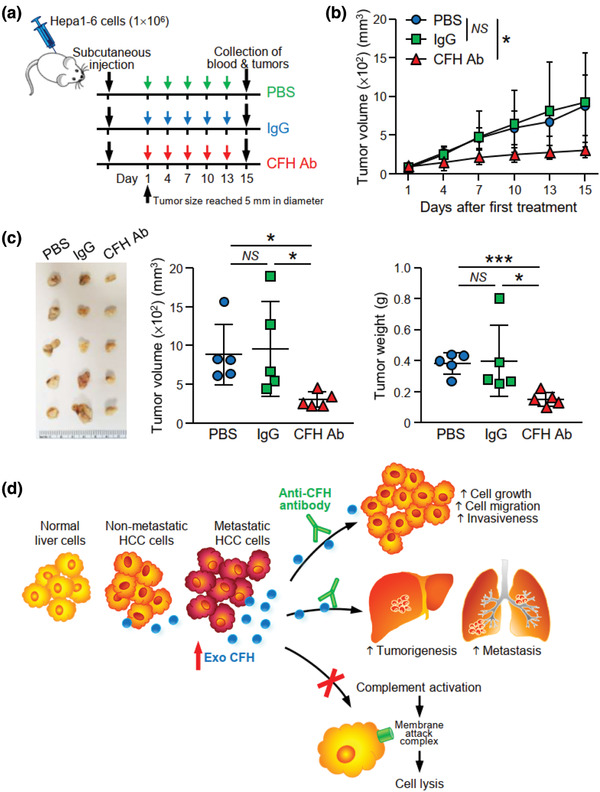
Anti‐CFH antibody treatment suppresses tumour development in syngeneic mouse model. (a) Schedule of anti‐CFH antibody administration in mice. Hepa1‐6 cells were injected subcutaneously into C57BL/6N mice. Administration of antibody was started when the tumour size reached 5 mm in diameter. PBS, control IgG or murine anti‐CFH antibody (200 μg/mouse) were administered by intraperitoneal injection once every 3 days for 2 weeks. (b) Tumour size was measured regularly during the experimental period and plotted. (c) Tumours developed were excised and tumour size and weight were measured. Image showing the fixed tumours (*left*). Tumour volume (*middle*) and weight (*right)* were plotted. (d) A proposed model illustrating the role of EV‐CFH in HCC. EVs with higher CFH level are derived from HCC cells with higher metastatic potential when compared to EVs of non‐metastatic HCC cells and normal liver cells. EV‐CFH promotes HCC cell growth and motility in culture and tumorigenesis and metastasis in animals. Uptake of EVs also protects HCC cells from complement‐mediated cytotoxicity, thus facilitating the survival and growth of cells in liver and distant lung tissues. Data are represented as mean ± SEM. ^*^ *P* < 0.05, ^***^ *P* < 0.001. *P* < 0.05 is considered as statistically significant. *NS*, not significant

With the use of a recombinant anti‐CFH antibody which has been shown to cause complement activation (Bushey et al., 2016), the effect of MHCC97L‐EVs on HCC cell migration and invasion in the presence of a specific anti‐CFH antibody was studied. Anti‐CFH antibody significantly reduced migration and invasion of MIHA and PLC/PRF/5 cells induced by MHCC97L‐EVs (Figure S4). In an EV education model, co‐injection of MHCC97L‐EVs with anti‐CFH antibody but not with control IgG abrogated the enhancement of liver tumour formation induced by MHCC97L‐EVs (Figure 7a‐b). As shown in Figure 5d, MHCC97L‐EVs promoted the colonization of murine p53‐/‐;Myc hepatocytes in the lungs. Co‐injection of EVs and murine cells with control IgG had no effect on the colonization of cells. However, the tumour promoting ability of MHCC97L‐EVs was significantly suppressed when co‐injected with anti‐CFH antibody (Figure 7c‐d). Ex vivo bioluminescence imaging and histological analysis revealed that the presence of metastatic lesions in the lungs was reduced in mice injected with anti‐CFH antibody (Figure 7e‐f). Finally, we examined the efficacy of anti‐CFH antibody in an immunocompetent mouse model in which Hepa1‐6 cells were subcutaneously injected followed by the administration of anti‐CFH antibody for 2 weeks (Figure 8a). Tumour development was delayed in mice injected with anti‐CFH antibody when compared to mice injected with PBS or control IgG (Figure 8b). Significant reductions in tumour volume and tumour weight were observed in the anti‐CFH antibody group compared to the PBS and control IgG groups (Figure 8c). These findings suggest a role of CFH in tumour formation and metastasis, which could be blocked by an anti‐CFH antibody (Figure 8d).

## 4 DISCUSSION

In this study, proteomic profiling of EVs derived from metastatic cells revealed significant alterations in a number of complement associated proteins when compared to EVs of normal liver cells, suggesting a potential role of the complement system in HCC progression and metastasis. Among these complement proteins, CFH was one of the uniquely expressed proteins in EVs of metastatic HCC cells. CFH is an abundant serum protein, and has an important role in host defence (Parente, Clark, Inforzato, & Day, 2017). Its primary role is to regulate the alternative pathway of the complement system, ensuring that the complement system is targeting pathogens and unwanted cells, but does not damage normal host cells. Unlike the classical and lectin pathways, which are dependent on the immune complexes and carbohydrate ligands on the surface of microorganisms for their initiation, the alternative pathway occurs due to the spontaneous hydrolysis of C3 (Chen, Daha, & Kallenberg, 2010). Nevertheless, the alternative pathway is tightly regulated by cell surface‐associated regulators such as complement receptor 1 (CR1), membrane cofactor protein (CD46), and decay‐accelerating factor (CD55) as well as the fluid phase regulator, CFH (Łukawska, Polcyn‐Adamczak, & Niemir, 2018). CFH is a large soluble glycoprotein, typically produced in the liver and is one of the most abundant plasma proteins. It has been suggested that interaction between CFH and C3 facilitates the cleavage of C3 (Soames & Sim, 1997). Intriguingly, the CFH‐C3 interaction possibly exists in EVs as both CFH and C3 are highly expressed in EVs of metastatic HCC cells. CFH not only functions in the fluid phase but acts on the extracellular matrix and host cell surface through its binding recognition domain with polyanions (Blaum et al., 2015; Clark et al., 2006). Furthermore, CFH limits inflammation in the atherosclerotic lesions via its interaction with apoliporotein E (apoE) leading to the reduction of apoE concentration (Nissilä et al., 2018). This coincides with our proteomic profiling that apoE is not found in MHCC97L‐EVs in which CFH is highly expressed but is found in MIHA‐EVs in which CFH is not expressed. In the context of cancers, cancer cells have been shown to make use of CFH to reduce complement activation in their microenvironment (Wilczek et al., 2008). A study in lung cancer has shown tumour cells that express CFH can prevent complement activation in vivo and protect tumour cells from complement‐mediated cytolysis, thereby promoting tumour development (Ajona, Hsu, Corrales, Montuenga, & Pio, 2007).

Autoantibodies to CFH in cancer were first discovered in the blood of patients with early stage, non‐metastatic non‐small cell lung cancer (NSCLC) (Amornsiripanitch et al., 2010). Anti‐CFH antibodies purified from these NSCLC patients recognize a conformationally distinct form of CFH, when bound to the tumour cell surface. The autoantibody binds to a very specific functional domain of CFH in SCR 19, blocks binding of CFH to C3b on the tumour cell, and thus promotes complement‐dependent tumour cell lysis with subsequent inhibition of tumour growth (Bushey et al., 2016). These findings suggest that the high expression of CFH in cancer cells inhibits the attack of the complement system on cancer cells, which is beneficial for tumour cell survival and progression.

Our findings demonstrate the effect of CFH in promoting HCC cell growth and motility. These data suggest that CFH expression can potentially contribute to the enhanced aggressive phenotypes of cells. Further investigation is needed to fully understand how CFH regulates HCC cell growth and motility. Another study showed that CFH increase the expression of Nanog, Oct4, Sox2 and c‐Myc, and induces stemness features of HCC cells (Seol et al., 2016). Increased expression of CFH has been reported in lung cancer, cutaneous squamous cell carcinoma and HCC (Riihilä et al., 2014; Yoon, Hwang, & Sung, 2019). Overexpression of CFH is associated with poorer overall survival of cancer patients (Laverdière et al., 2016), and is suggested to be a prognostic biomarker. Nevertheless, complement proteins have also been shown to promote cancer‐related inflammation that is conducive to tumour growth. In a chemically induced carcinogenesis model of PTX3‐deficient mice, defective recruitment of CFH to cancer cells results in higher levels of the anaphylatoxins C3a and C5a due to complement activation. A high level of anaphylatoxins leads to an exacerbated inflammatory response and enhanced carcinogenesis (Bonavita et al., 2015). A CFH‐deficient mouse model revealed an elevation in inflammatory signalling pathways and an increase in spontaneous tumour formation in the liver (Laskowski et al., 2020). Plasma CFH also suppresses angiogenesis by reducing the migration of endothelial cells (Liu & Hoh, 2017). These studies indicate that CFH may also play a dual role in tumorigenesis.

EVs confer the stability of their cargo and ensure the delivery of cargo to distant sites (Jeyaram & Jay, 2017). Thus, the inclusion of CFH in secreted EVs appears to enable distant intercellular communications. The level of EV‐CFH correlated with the metastatic potential of cells from which the EVs are released, implicating the role of EV‐CFH in HCC progression and metastasis. Functionally, we demonstrated the ability of EV‐CFH to reduce complement‐mediated cell lysis and promote tumour growth and colonization of cells to the lungs. Our study suggests that cancer cells hijack the complement system by their released EVs as an escape mechanism from immunosurveillance, thereby contributing to HCC tumorigenesis and metastasis. It is noted that besides CFH, other complement associated proteins are also significantly altered in the EVs of metastatic HCC cells. Nevertheless, how these proteins make a concerted effort in the regulation of complement system is uncertain. These differentially expressed complement proteins are worthy of further investigation concerning their involvement in tumorigenesis.

Several existing therapeutic strategies have an effect on the complement system. For instance, antibody therapy using a combination of cetuximab and matuzumab induces strong complement activation, leading to complement‐mediated lysis of squamous cell carcinoma and glioblastoma cells (Dechant et al., 2008). An *in vivo* study demonstrated that the significant effect of cetuximab in suppressing A549 lung tumour growth is abrogated in mice with complement depletion, suggesting that the activity of cetuximab is not only caused by its ability to bind and block EGFR, but is driven by complement activation (Hsu et al., 2010). In this study, we revealed that blocking CFH‐enriched EVs using anti‐CFH antibody delayed tumour development and metastasis. In accordance with our study, knockdown of CFH in A549 cells potentiates the antitumor effect of cetuximab (Hsu et al., 2010). In conclusion, our study reveals a fundamental role of EV‐CFH released by HCC. The potential to target CFH‐enriched EVs can be exploited as a novel therapeutic strategy to enhance treatment options of HCC.

## CONFLICT OF INTEREST

The authors report no conflict of interest. E.F.P. is a co‐founder and CEO of Grid Therapeutics.

## AUTHOR CONTRIBUTIONS

Xiaowen Mao and Longyin Zhou performed most of the experiments and analysed data. Angel Po Yee Ma performed the syngeneic mouse model. Cherlie Lot Sum Yeung provided technical assistance throughout the study. Tung Him Ng, Samuel Wan Ki Wong and Sze Keong Tey characterized the isolated EVs. Bonnie Hei Man Liu assisted in manuscript preparation. Yi Man Eva Fung performed mass spectrometry. Peihua Cao is involved in experimental design. Edward F. Patz provided resources and suggestions in experimental design, and assisted in writing the manuscript. Yi Gao coordinated the study. Judy Wai Ping Yam coordinated and supervised the project, wrote manuscript and provided funds.

## Supporting information



Supporting Information

Supporting Information

Supporting Information

## Data Availability

All data generated or analysed during this study are included in this published article (and its supplementary information files).
