# Supplementary material for: Tumour extracellular vesicle‐derived Complement Factor H promotes tumorigenesis and metastasis by inhibiting complement‐dependent cytotoxicity of tumour cells
Source: J Extracell Vesicles. 2020 Nov 28;10(1):e12031. doi: 10.1002/jev2.12031 (PMC7890557; doi:10.1002/jev2.12031)
Supplement: Supplementary file 1 — Supporting Information [file JEV2-10-e12031-s001.docx]

**Tumor extracellular vesicle-derived Complement Factor H promotes tumorigenesis and metastasis by inhibiting complement-dependent cytotoxicity of tumor cells**

Xiaowen Mao^1,11^, Longyin Zhou^1,11^, Sze Keong Tey^1^, Angel Po Yee Ma^1^, Cherlie Lot Sum Yeung^1^, Tung Him Ng^1^, Samuel Wan Ki Wong^1^, Bonnie Hei Man Liu^1^, Yi Man Eva Fung^2^, Edward F. Patz Jr.^3,4^, Peihua Cao^5,6^, Yi Gao^6,7,8,9^, Judy Wai Ping Yam^6,1,10^

**Supplementary Information**

**Supplementary Materials and Methods**

**Colony formation assay**

Cells pretreated with EVs for 72 h were seeded at a density of 1×10^3^ - 5×10^3^ per well in 6-well plates in triplicates and incubated for 1-2 weeks. After incubation, the colonies formed were fixed, stained with crystal violet (USB) and counted.

**Soft agar assay**

The 60-mm culture plate was first coated with bottom agar (1% agarose, 2× DMEM, 20% FBS and 2% penicillin and streptomycin). After the bottom layer has solidified, top agar (0.4% agarose, 2× DMEM, 20% FBS and 2% penicillin and streptomycin) containing 1×10^4^ EV-pretreated cells were overlaid on top of the bottom agar. The experiment was performed in triplicates. After the top agar has solidified, the culture plates were sealed with parafilm and incubated at 37°C for 3-4 weeks. At the end of experiment, 4 fields per well were randomly selected and photographed under a microscope connected with a CCD camera (Nikon). The number of colonies was counted.

**Cell migration assay**

Cell motility was performed using Transwell® Permeable Support assay (with inserts of 6.5 mm in diameter) (Corning) according to the manufacturer’s instructions. Briefly, EV-pretreated cells were suspended in serum-free medium and added to the upper chamber, while the lower chamber was filled with culture medium supplemented with either fetal bovine serum or hepatocyte growth factor which acts as a chemo-attractant. Cells were allowed to migrate through the membrane for 16-18 h. After incubation, the cells which remained within the upper chamber were removed and the medium in the upper chamber was discarded. The migrated cells were fixed and stained with crystal violet. Four fields per well were randomly selected and photographed. The number of migrated cells was counted.

**Cell invasion assay**

Cell invasion assay was performed using Transwell® Permeable Support assay (Corning) coated with BD Matrigel™ Basement Membrane Matrix (BD Biosciences) according to the manufacturer’s guidelines. The transwells coated with matrigel were incubated at 37°C for at least 30 min before being used for invasion assay. The EV-pretreated cells were suspended in serum-free medium and seeded into the matrigel-coated upper chamber. The lower chamber was filled with medium supplemented with either fetal bovine serum or hepatocyte growth factor. After incubation for 16-18 h, the invaded cells were fixed and stained with crystal violet. The number of invaded cells in 4 randomly selected fields per well were photographed and counted.

**Western blot analysis**

The membrane was blocked with 5% non-fat milk in Tris-buffered saline containing 0.1% Tween 20 (TBST) at room temperature for 1 h. After blocking, the membrane was incubated overnight with primary antibody diluted in 5% non-fat milk or BSA in TBST at 4°C. After incubation, the membrane was washed 3 times with TBST followed by 1 hr incubation with the secondary antibody conjugated with horseradish peroxidase diluted in 5% non-fat milk/TBST. After incubation, the membrane was washed with TBST. The signal of membrane was detected using ECL™ Western Blotting Detection Reagents (GE Healthcare). The following antibodies were used in the study: primary antibodies include anti-CFH (R&D Systems), anti-TSG101 (BD Biosciences), anti-CD9 (Abcam), anti-CD63 (Abcam), anti-Alix (Santa Cruz), anti-p62 (Abcam), anti-GM130 (Abcam) and anti-β-actin (Sigma-Aldrich). Secondary antibodies include anti-mouse, anti-rabbit and anti-goat IgG (H+L), HRP conjugate (Thermo Fisher).

**Immunogold labeling of EVs**

Purified EVs suspended in PBS were allowed to absorb for 20 min on formvar-carbon coated nickel grids under a dry environment. After saturating non-specific binding sites with PBS containing 1% BSA, the EVs were labelled with goat anti-CFH antibody and rabbit anti-CD63 antibody, followed by anti-goat antibody-15 nm gold particle and anti-rabbit antibody-5 nm gold particle, respectively. Immuno-complexes were then cross-linked with 1% glutaraldehyde and the samples were washed in distilled water, contrasted and embedded. The samples were viewed under Philips CM100 Transmission Electron Microscope and images were taken.

**Enzyme-Linked Immunosorbent Assay (ELISA)**

The expressions of CFH and CD63 in EVs were detected using CFH (Human) ELISA Kit (Abnova) and Human CD63 ELISA Kit (Aviva Systems Biology), respectively. EVs collected from the conditioned medium of cell lines or blood samples were diluted using EIA Diluent solution. The diluted sample was added to each well of a 96-well plate and incubated for 2 h. After incubation, the wells were washed. The biotinylated anti-CFH antibody was added to each well and incubated for 1 h. The wells were washed after incubation and incubated with 1:100 SP Conjugate for 30 min. After incubation, the wells were washed and added with Chromogen Substrate. The reaction was terminated with the addition of Stop Solution. The reading was measured at 450 nm using the Infinite® F200 microplate reader (Tecan). The concentration of CFH was deduced from the standard curve.

**Quantitative real-time polymerase chain reaction (qPCR)**

RNA was extracted from cells using TRIzol™ Reagent (Invitrogen). Extracted RNA was mixed with SuperScript™ VILO™ MasterMix (Invitrogen) and subjected to reverse transcription. The complementary DNA obtained were examined for CFH expression using qPCR. Absolute copies of CFH were determined by normalization with the housekeeping gene hypoxanthine guanine phosphoribosyltransferase (HPRT). The primers used for qPCR are listed in Supplementary Table S6. The reaction mixture containing 30 ηg cDNA, 10 μl 2× SYBR Green Mix (Integrated DNA Technologies), 5 μM forward primer and 5 μM reverse primer were subjected to PCR in LightCycler® 480 Instrument II (Roche Molecular Systems).

**Supplementary Tables**

**Supplementary Table S1. Results of mass spectrometry produced by MaxQuant software** (Attached as separate excel file).

**Supplementary Table S2. Common and unique proteins identified in EVs of MIHA and MHCC97L cells** (Attached as separate excel file).

**Supplementary Table S3. The pathways that are associated with proteins found in MHCC97L-EVs.**

| **Pathway Category** | **Count** | ***p*-value** | **Bonferroni** | **Benjamini** | **FRD** |
| --- | --- | --- | --- | --- | --- |
| hsa04512: ECM-receptor interaction | 15 | 8.17E-12 | 1.24E-09 | 1.24E-09 | 9.78E-09 |
| hsa04510: Focal adhesion | 20 | 2.98E-11 | 4.54E-09 | 2.27E-09 | 3.57E-08 |
| **hsa04610: Complement and coagulation cascades** | **9** | **4.12E-06** | **6.25E-04** | **2.09E-04** | **4.93E-03** |
| hsa05130: Pathogenic Escherichia coli infection | 8 | 5.41E-06 | 8.22E-04 | 2.05E-04 | 6.47E-03 |
| hsa05146: Amoebiasis | 10 | 1.36E-05 | 2.06E-03 | 4.13E-04 | 1.63E-02 |
| hsa05322: Systemic lupus erythematosus | 9 | 4.92E-04 | 7.21E-02 | 1.24E-02 | 5.89E-01 |
| hsa04151: PI3K-Akt signaling pathway | 14 | 8.44E-04 | 1.20E-01 | 1.82E-02 | 1.01 |
| hsa04145: Phagosome | 9 | 1.04E-03 | 1.46E-01 | 1.95E-02 | 1.23 |
| hsa05205: Proteoglycans in cancer | 9 | 6.20E-03 | 6.11E-01 | 9.97E-02 | 7.17 |
| hsa05150: Staphylococcus aureus infection | 5 | 6.37E-03 | 6.21E-01 | 9.26E-02 | 7.37 |
| hsa05134: Legionellosis | 5 | 6.37E-03 | 6.21E-01 | 9.26E-02 | 7.37 |
| hsa04974: Protein digestion and absorption | 6 | 7.18E-03 | 6.66E-01 | 9.48E-02 | 8.27 |
| hsa00010: Glycolysis / Gluconeogenesis | 5 | 1.35E-02 | 8.73E-01 | 1.58E-01 | 15.00 |
| hsa05145: Toxoplasmosis | 6 | 1.77E-02 | 9.34E-01 | 1.89E-01 | 19.26 |
| hsa05100: Bacterial invasion of epithelial cells | 5 | 2.24E-02 | 9.68E-01 | 2.18E-01 | 23.74 |
| hsa05222: Small cell lung cancer | 5 | 2.96E-02 | 9.90E-01 | 2.62E-01 | 30.17 |
| hsa05144: Malaria | 4 | 2.98E-02 | 9.90E-01 | 2.49E-01 | 30.34 |
| hsa04540: Gap junction | 5 | 3.30E-02 | 9.94E-01 | 2.59E-01 | 33.08 |
| hsa04611: Platelet activation | 6 | 3.35E-02 | 9.94E-01 | 2.50E-01 | 33.48 |

The number of proteins in each pathway (Count), *p*-value, Bonferroni and Benjamini corrected values and the false discovery rate (FRD) are analyzed by DAVID v6.8 pathway program (KEGG_PATHWAY).

**Supplementary Table S4. Proteins associated with complement system that only expressed in EVs of MHCC97L cells**

| *Complement proteins* | | *Expression level* |
| --- | --- | --- |
| **CFH** | **Complement Factor H** | **4.912 × 10^8^** |
| C1R | Complement C1r subcomponent | 4.168 × 10^7^ |
| SERPINE1 | Plasminogen activator inhibitor 1 | 7.367 × 10^7^ |
| C1S | Complement C1s subcomponent | 1.769 × 10^7^ |
| C8B | Complement C8 beta chain | 9.249 × 10^6^ |

**Supplementary Table S5. Complement proteins upregulated in MHCC97L-EVs when compared to EVs of normal liver cells**

| *Complement proteins* | | *Expression level* | *Fold enhanced* |
| --- | --- | --- | --- |
| C4A, C4B | C4a, C4b anaphylatoxin | 4.912 × 10^8^ | 17.6 |
| C1QTNF3 | Complement C1q tumor necrosis factor-related protein 3 | 1.769 × 10^7^ | 16.2 |

**Supplementary Table S6. Sequence of primers for RT-qPCR**

| **Oligo name** | **Sequence (5’ to 3’)** |
| --- | --- |
| CFH-F | GTGAAGTGTTTACCAGTGACAGC |
| CFH-R | AACCGTACTGCTTGTCCAAAA |
| HPRT-F | CCTGGCGTCGTGATTAGTGAT |
| HPRT-R | AGACGTTCAGTCCTGTCCATAA |

**Supplementary Figures**


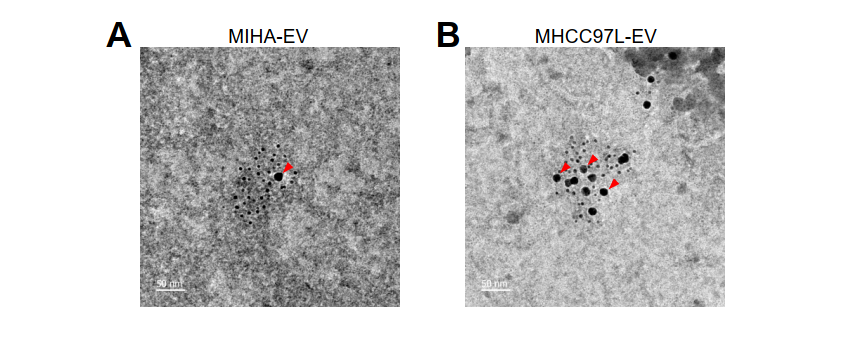


**Supplementary Figure S1.** **Detection of higher CFH expression on EVs of MHCC97L cells.** Representative electron micrograph of EVs of MIHA (A) and MHCC97L (B) cells subjected to immunogold labeling using anti-CD63 and anti-CFH antibodies coupled to 5- and 15-nm gold particles, respectively. Arrowhead indicates the 15-nm gold particles of CFH. Scale bar: 50 nm.


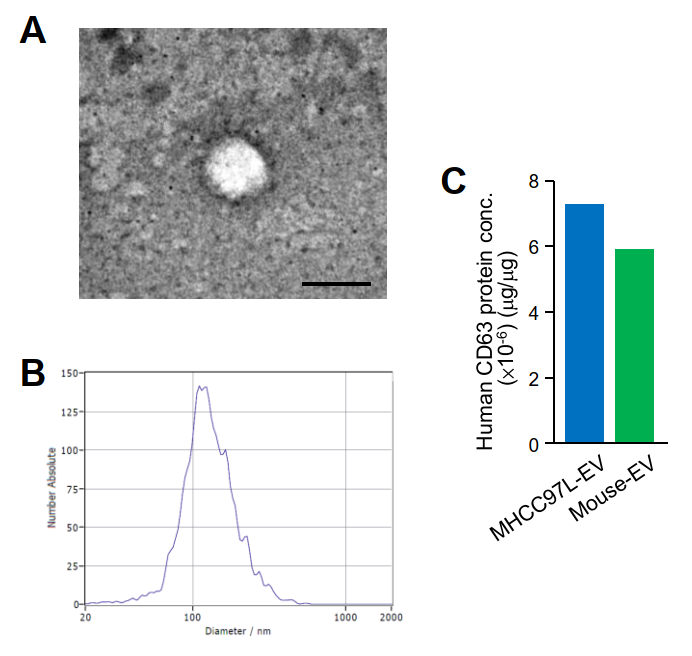


**Supplementary Figure S2. Characterization of EVs extracted from serum of mouse implanted with MHCC97L tumor seed.** (A) Representative electron micrograph of circulating EVs isolated from serum of mouse collected at week 5 post implantation of MHCC97L tumor seed. (B) Size distribution of mouse EVs measured by nanoparticle tracking analyzer. (C) Determination of human CD63 expression level in circulating EVs obtained from serum of a MHCC97L tumor bearing mouse at week using ELISA. MHCC97L-EV is included as a control.


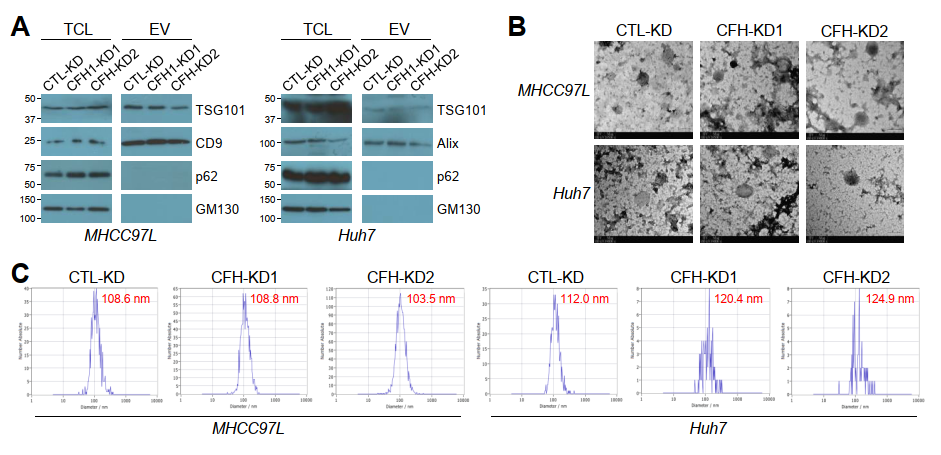


**Supplementary Figure S3.** **Characterization of the isolated EVs.** (A) Western blot analysis of EV molecular markers in 30 ug of total cell lysate (TCL) and 15 ug of EV derived from the non-target control (CTL-KD) and CFH knockdown (CFH-KD1 and CFH-KD2) cells established in MHCC97L (*left*) and Huh7 (*right*). Positive EV markers TSG101, CD9 and Alix, and negative EV markers nucleoporin p62 and cis-Golgi marker GM130 were examined. (B) Representative transmission electron micrographs of EVs isolated from the conditioned medium of control and CFH knockdown cells are shown. Scale bar: 100 nm. (C) Size distribution of the indicated EVs measured by nanoparticle tracking analyzer. Median particle size of EVs is indicated.


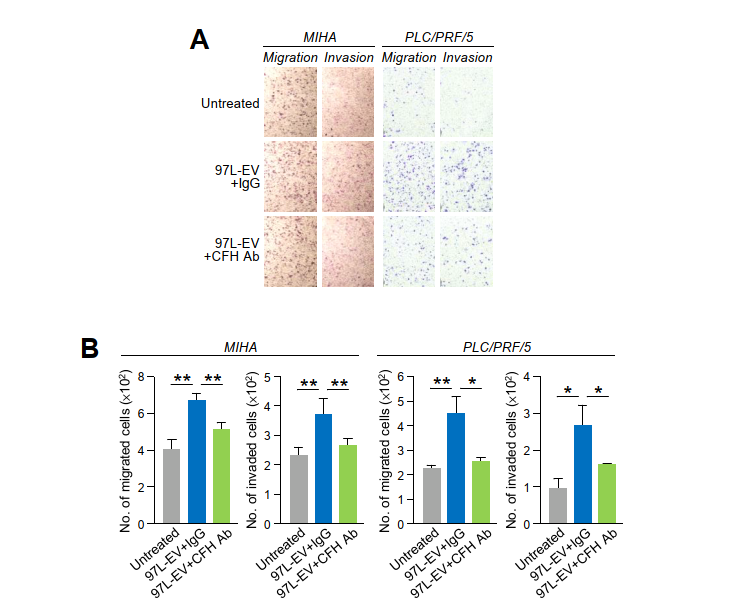


**Supplementary Figure S4.** Anti-CFH antibody diminishes the promoting effect of MHCC97L EVs in cell migration and invasiveness. MIHA and PLC/PRF/5 cells were treated with EVs derived from metastatic MHCC97L (97L-EV) cells in the presence of 250 μg/ml control IgG or anti-CFH antibody. After incubation for 72 h, cells were subjected to migration and invasion assays. (A) Representative images of fixed and crystal violet stained migrated and invaded cells. (B) Numbers of migrated and invaded cells were plotted. Data are represented as mean ± SEM. **P* < 0.05, ***P* < 0.01. *P* < 0.05 is considered as statistically significant.
